# Protonated nanostructured aluminosilicate (NSAS) reduces plasma cholesterol concentrations and atherosclerotic lesions in Apolipoprotein E deficient mice fed a high cholesterol and high fat diet

**DOI:** 10.1186/1476-511X-8-30

**Published:** 2009-07-28

**Authors:** Olena Sivak, Jerry Darlington, Pavel Gershkovich, Panayiotis P Constantinides, Kishor M Wasan

**Affiliations:** 1 Division of Pharmaceutics and Biopharmaceutics, Faculty of Pharmaceutical Sciences, The University of British Columbia, Vancouver, British Columbia, Canada; 2 AMCOL International Corporation, Hoffman Estates, Illinois, USA; 3 Biopharmaceutical and Drug Delivery Consulting LLC, Gurnee, Illinois, USA

## Abstract

The aim of this work was to assess the effect of chronic administration of protonated nanostructured aluminosilicate (NSAS) on the plasma cholesterol levels and development of atherosclerotic lesions in Apolipoprotein (ApoE) deficient mice fed a high cholesterol and high fat diet. Apolipoprotein E (ApoE) deficient mice were divided into the following treatment groups: protonated NSAS 1.4% (w/w), untreated control and 2% (w/w) stigmastanol mixed with high-cholesterol/high-fat diet. Animals were treated for 12 weeks, blood samples were withdrawn every 4 weeks for determination of plasma cholesterol and triglyceride levels. At the end of the study the aortic roots were harvested for assessment of atherosclerotic lesions. NSAS at 1.4% (w/w) and stigmastanol at 2% (w/w) treatment groups showed significant decreases in plasma cholesterol concentrations at all time points relative to the control animals. The lesion sum area in 1.4% (w/w) NSAS and 2% (w/w) stigmastanol groups were significantly less from the control animals. In conclusion, in this study, the effectiveness of chronic administration of protonated NSAS material in the reduction of plasma cholesterol levels and decrease in development of atherosclerotic lesions was demonstrated in Apo-E deficient mice model.

## Introduction

**Figure 1:**
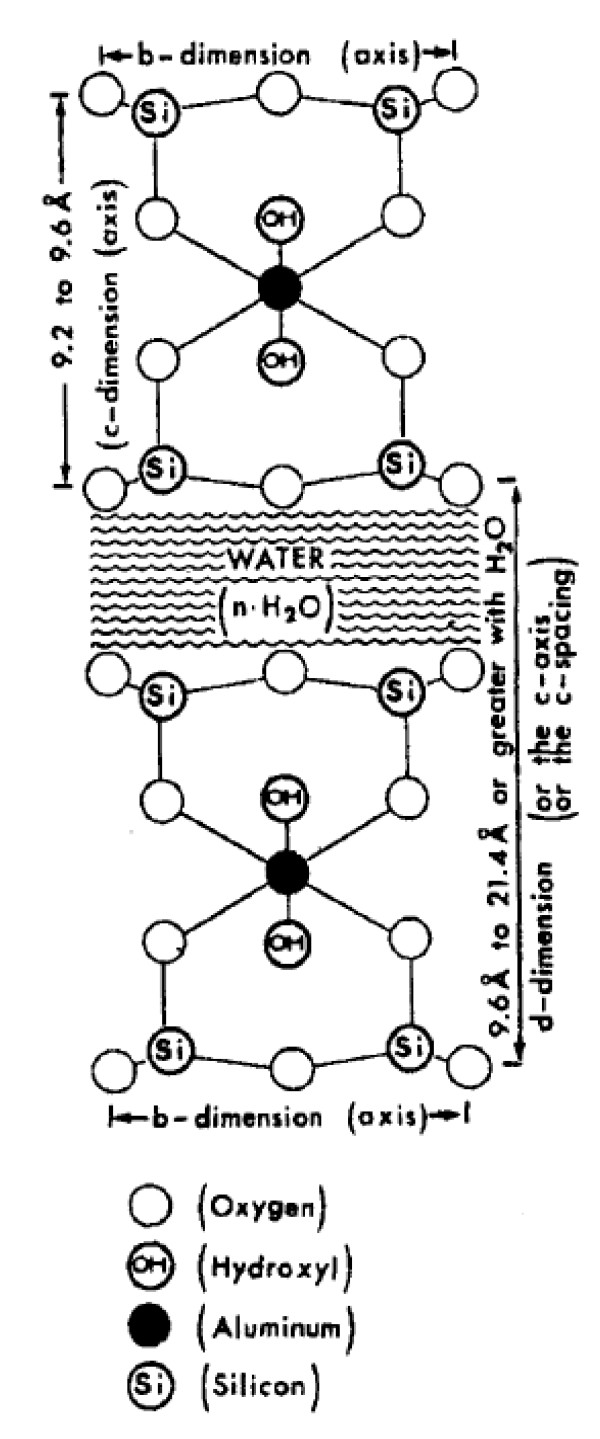
**Schematic presentation of montmorillonite structure**. Montmorillonite is a layered silicate with the property of adsorbing organic substances on its external surfaces or within its interlaminar space. The hydration of the clay induces swelling, which is mostly attributed to the increase in d (or c) dimension.

Elevated plasma cholesterol levels have been associated with increased risk of atherosclerosis and coronary artery disease [1]. Although competitive inhibitors of 3-hydroxy-3-methylglutaryl coenzyme A (HMG-CoA) reductase (statins) are the gold standard in the treatment of hypercholesterolemia, there is a long history of use of cholesterol absorption inhibitors as an alternative/adjuvant method of treating hypercholesterolemia [1]. The main agents are plant sterols, plant stanols and ezetimibe. However, since these agents are reported to be absorbed into blood circulation, there is a potential for systemic adverse effects [2-5]. Potential candidates for non-absorbable agents for inhibiting gastrointestinal absorption of cholesterol would be the naturally occurring aluminosilicates clays (Figure 1) [6]. A calcium montmorillonite clay has been reported to be safe and effective in reducing of exposure to aflatoxin by adsorption mechanism in rodents and humans [7,8]. We have previously reported that protonated nanoscaled aluminosilicate compound efficiently inhibited the intestinal absorption of cholesterol following acute administration in a rat model [[9]; See Additional File 1]. It was unclear, however, what will be the effect of NSAS in the chronic administration on plasma cholesterol levels and on development of pathophysiology of atherosclerosis [10]. Thus, the current study was designed to elucidate the effect of chronic administration of protonated form of NSAS to Apolipoprotein-E (Apo-E) deficient mice on plasma cholesterol concentrations and formation of aortic atherosclerotic lesions.

## Methods

Mice, C57B1/6 B6.129P2-ApoE™1UNC, 4 week old, with homozygous deletion of the ApoE gene (apolipoprotein E knock-out) were purchased from Jackson Laboratories, USA. The Apo-E deficient mice model has been used extensively, since these mice develop severe hypercholesterolemia and atherosclerotic lesions similar to those observed in humans [10,11].

The protonated NSAS material was prepared as previously reported [[9], See Additional File 1]. Briefly, the crude NSAS was dried to 10% moisture content and the particle size was reduced by a passing through a 200 mesh (74 μm) screen. The base NSAS was then purified by a previously reported method [12] and mixed with deionized water using a blender at 11,500 rpm for 5 min. As a result of the purification process essentially all of the exchangeable surface cations were replaced by sodium ions. Sodium NSAS sample was pumped through two lab-scale ion-exchange columns filled with hydrogen-loaded resin exchange beads to protonate the montmorillonite.

All animals used in this study were cared for in accordance with the principles promulgated by the Canadian Council in Animal Care and the University of British Columbia. The research adhered to the "Principles of Laboratory Animal Care" (NIH publication #85-23, revised in 1985).

The Apo-E deficient mice (Jackson Laboratories, USA) were divided into the following treatment groups: protonated NSAS 1.4% w/w, untreated control and 2% w/w stigmastanol. All animals received high-cholesterol/high-fat diet (45 kcal% fat) (Research Diets Inc., USA) for the duration of the study. The tested active compounds were incorporated into the diet. All animals were treated for 12 weeks. Blood samples were withdrawn from saphenous vein every 4 weeks and at the end of the study for determination of total plasma cholesterol and triglyceride levels. At the end of the study the animals were sacrificed and aortic arch was harvested for histopathology assessment of atherosclerotic lesions in control animals and in groups that showed statistically significant differences in their plasma lipid profile relatively to the control group. Tissue surrounding the aorta including all fat were trimmed, and frozen in liquid nitrogen. The aorta was cut in 3 consecutive slices (10 μmol/L) 5 mm above the aortic root. Slides were stained with Oil-Red-O, Movat's Pentachrome and Hematoxylin-eosin. An independent pathologist, blinded to the treatment groups scored the lesion formation based on cumulative atherosclerotic exposure area (sum area).

Statistical analyses were performed using one-way ANOVA followed by Dunnett multiple comparisons test. Results were expressed as mean +/- SEM, p < 0.05 indicated a significant difference between groups.

## Results and discussion

**Figure 2:**
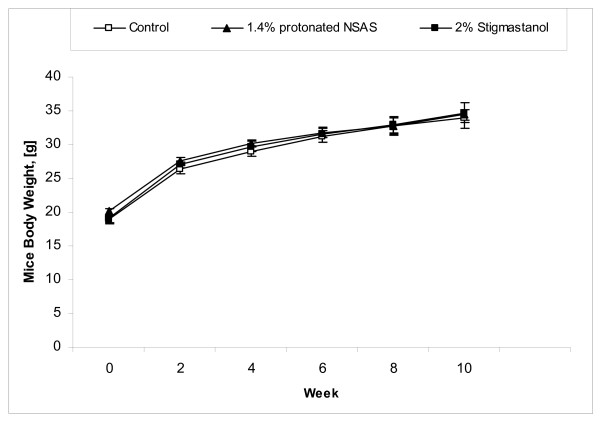
**Body weight (Mean +/- SEM) from the beginning of the treatment (week 0) in the Apo-E deficient mice treated with 1.4% w/w (n = 8) of protonated NSAS, 2% w/w stigmastanol (n = 7) vs. untreated control (n = 8)**. No statistically significant differences between treatment and untreated control groups were observed.

**Figure 3:**
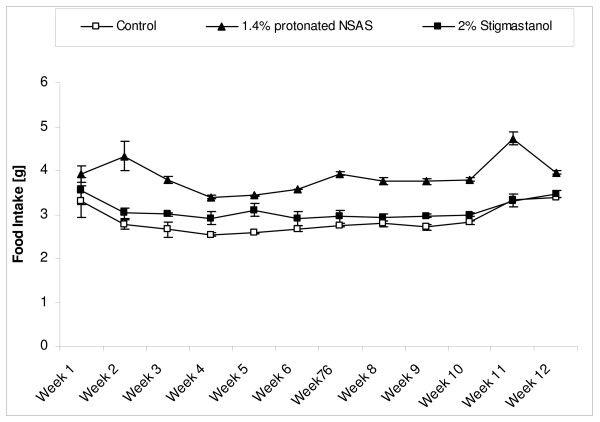
**Weekly food in-take (Mean +/- SEM) from the beginning of the treatment (week 0) in the Apo-E deficient mice treated with 1.4% w/w (n = 8) of protonated NSAS, 2% w/w stigmastanol (n = 7) vs. untreated control (n = 8)**. No statistically significant differences between treatment and untreated control groups were observed. SEM were not shown in this figure.

All animals based on physical appearance did not appear to have any deleterious effects from administration of NSAS at 1.4% w/w or stigmastanol at 2% w/w. The activity and behaviour of the animals were similar between all groups and consistent with the ApoE deficient phenotype. The baseline of the body weight was 19 g (average) and after 12 weeks was 33.2 (average). As a result of similar food and water consumption (data not shown) during all period of study, the body weight increased by the same amount over the experimental period in all groups (treatment and control) (Figure 2). The average food intake for all treatment and control groups throughout the duration of study was 3.14 g per day (Figure 3).

**Figure 4:**
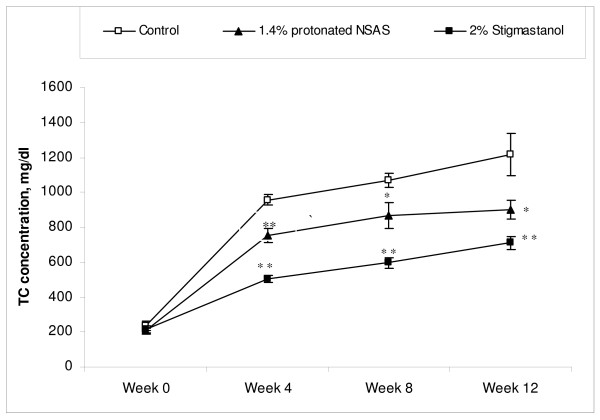
**Total plasma cholesterol concentrations (Mean +/- SEM) from the beginning of the treatment (week 0) to the end of treatment (week 12) in the Apo-E deficient mice treated with 1.4% w/w (n = 8) of protonated NSAS, 2% w/w stigmastanol (n = 7) vs. untreated control (n = 8)**. Statistically significant differences for both 1.4% w/w protonated NSAS and 2% w/w stigmastanol at 4, 8 and 12 weeks compared to untreated controls (*p < 0.05 vs. untreated controls and **p < 0.01 vs. untreated controls) were observed; one-way ANOVA followed by Dunnett multiple comparisons test

**Figure 5:**
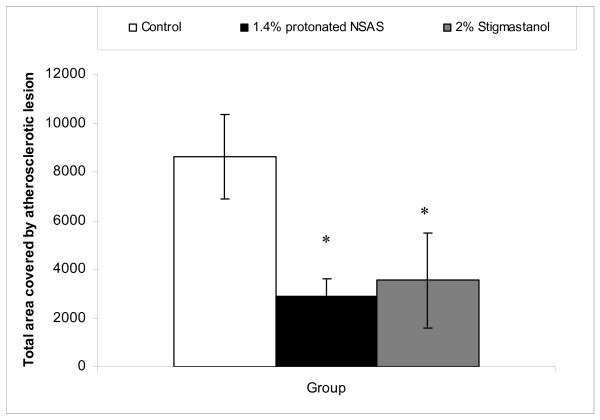
**Total area covered by atherosclerotic lesion (Mean +/- SEM) in ApoE-deficient mice treated with 1.4% w/w (n = 8) of protonated NSAS, 2% w/w stigmastanol (n = 7) vs. untreated control (n = 8)**. Statistically significant differences for both 1.4% protonated NSAS and 2% w/w stigmastanol compared to untreated controls (*p < 0.05 vs. untreated controls) were observed; one-way ANOVA followed by Dunnett multiple comparisons test.

**Figure 6:**
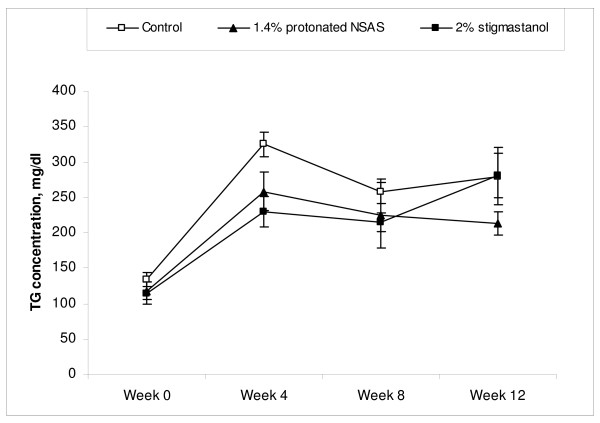
**Total plasma triglyceride concentrations (Mean +/- SEM) from the beginning of the treatment (week 0) to the end of treatment (week 12) in the Apo-E deficient mice treated with 1.4% w/w (n = 8) of protonated NSAS, 2% w/w stigmastanol (n = 7) vs. untreated control (n = 8)**. No statistically significant differences between treatment and untreated control groups were observed.

The total plasma cholesterol concentrations through duration of the study in different treatment groups are shown in Figure 4. The treatment by protonated NSAS at 1.4% w/w and stigmastanol at 2% w/w resulted in a significant decrease in plasma cholesterol levels at 4, 8 and 12 weeks of the study compared to untreated controls. This cumulative reduction in plasma cholesterol levels over the duration of the study is reflected by a statistically significant difference in histopahotogically evaluated total area covered by atherosclerotic lesion in the 1.4% w/w NSAS and 2% w/w stigmastanol treatment groups compared to untreated controls (Figure 5). The change in plasma triglyceride levels through duration of the study in different treatment groups is shown in Figure 6. There are no statistically significant differences in total triglyceride levels as well as in change in triglyceride levels from the baseline between all treatment groups, at all time points, relatively to the control animals. This observation, in addition to the fact that there were no changes in body weight (Figure 2) and food intake (Figure 3) throughout the study indicates that it is unlikely that protonated NSAS compound affects significantly the intestinal absorption of triglyceride which is the main component of lipids in the diet. When the food intake in figure 3 is plotted as cumulative intake over time for each group no statistically significant differences are observed (data not shown). Thus the effect we observe in Figure 4 (plasma cholesterol levels) and Figure 5 (atherosclerotic lesion area) is due to the effect of the compounds.

In conclusion, in this study we have demonstrated the effect of protonated NSAS material on reducing plasma cholesterol concentrations and the development of atherosclerotic lesions in Apo-E deficient mice model following chronic administration. The chronic administration of protonated NSAS material on cholesterol absorption seems not to affect the intestinal absorption of triglyceride, as demonstrated by absence of changes in plasma triglyceride concentrations and lack of differences in body weight and food intake. The shown significant effect of supplementation of high fat/high cholesterol diet with protonated NSAS material on the formation of atherosclerotic lesions is particularly important, since the final aim of cholesterol-lowering treatment is reduction of the development of atherosclerosis. Although the effect of protonated NSAS material on inhibition of cholesterol absorption following acute administration was demonstrated previously [[9], See Additional File 1], and the beneficial effect of chronic administration on plasma cholesterol levels and atherosclerotic lesions formation in Apo-E deficient mice was demonstrated in this study, the mechanism of cholesterol absorption inhibition is still not completely clear. In addition, a dose-response study with protonated NSAS would be required to confirm its activity. Future mechanistic studies aimed to elucidate the mechanism(s) by which the protonated form of NSAS material inhibits the intestinal absorption of cholesterol will be needed.

## Abbreviations

NSAS: nanostructured aluminosilicate; ApoE: apolipoprotein E; TC: total cholesterol; TG: triglyceride.

## Competing interests

The authors declare that they have no competing interests.

## Authors' contributions

OS carried out all aspects of the animal studies, including analysis of plasma lipids. JD participated in the design of the study and provided the NSAS material. PG participated in the design of the study and performed some of the aspects of the animal studies, data analysis and writing of the manuscript. PC participated in the design of the study. KW participated in the design of the study, data analysis and writing of the manuscript.

## Supplementary Material

Additional file 1 **Inhibition of Intestinal Absorption of Cholesterol by Surface-Modified Nanostructured Aluminosilicate Compounds**. This study demonstrated the ability of surface-modified nanostructured aluminosilicate (NSAS) compounds to reduce dietary cholesterol intestinal absorption in a rat model. Click here for file
