# Supplementary material for: Protonated nanostructured aluminosilicate (NSAS) reduces plasma cholesterol concentrations and atherosclerotic lesions in Apolipoprotein E deficient mice fed a high cholesterol and high fat diet
Source: Lipids Health Dis. 2009 Jul 28;8:30. doi: 10.1186/1476-511X-8-30 (PMC2727508; doi:10.1186/1476-511X-8-30)
Supplement: Additional file 1 — Inhibition of Intestinal Absorption of Cholesterol by Surface-Modified Nanostructured Aluminosilicate Compounds. This study demonstrated the ability of surface-modified nanostructured aluminosilicate (NSAS) compounds to reduce dietary cholesterol intestinal absorption in a rat model. [file 1476-511X-8-30-S1.pdf]

Inhibition of Intestinal Absorption of Cholesterol by Surface-Modified Nanostructured Aluminosilicate Compounds

PAVEL GERSHKOVICH,[1] JERRY DARLINGTON,[2] OLENA SIVAK,[1] PANAYIOTIS P. CONSTANTINIDES,[3] KISHOR M. WASAN[1]

[1]Division of Pharmaceutics and Biopharmaceutics, Faculty of Pharmaceutical Sciences, The University of British Columbia, Vancouver, British Columbia, Canada

[2]AMCOL International, Inc., Arlington Heights, Illinois 60004

[3]Biopharmaceutical and Drug Delivery Consulting LLC, Gurnee, Illinois 60031

Received 16 July 2008; revised 17 September 2008; accepted 29 September 2008

Published online 17 December 2008 in Wiley InterScience (www.interscience.wiley.com). DOI 10.1002/jps.21616

ABSTRACT: The aim of this work was to assess the ability of aqueous suspensions of surface-modified nanostructured aluminosilicate (NSAS) compounds to reduce the intestinal absorption of cholesterol in a rat model. The rats were divided into 10 treatment groups which included several NSAS compounds at various doses, ezetimibe at 10 mg/kg, stigmastanol at 50 mg/kg, and normal saline. All compounds and controls were independently administered by oral gavage and then a mixture of [^{3}H]cholesterol and cold cholesterol in 10% Intralipid® was immediately administered orally to the animals. Systemic blood was sampled and the concentration of cholesterol in plasma was determined by means of radioactivity. Protonation of NSAS using an ion-exchange column resulted in significant inhibition of cholesterol absorption relative to the control group (31.5% and 38.6% reduction in absorption of cholesterol for 50 and 100 mg/kg doses, respectively). Other surface-ion modifications of NSAS compounds did not show significant effect on intestinal cholesterol absorption. The inhibition of cholesterol absorption by ezetimibe was superior and by stigmastanol was equal to the effect of protonated NSAS in the doses investigated in this study. In conclusion, protonated NSAS material seems to inhibit significantly the intestinal absorption of dietary cholesterol in a rat model. © 2008 Wiley-Liss, Inc. and the American Pharmacists Association J Pharm Sci 98:2390–2400, 2009
Keywords: intestinal absorption; adsorption; biomaterials; colloid; lipids; gastrointestinal; inhibition; micelle; natural products; oral absorption

INTRODUCTION

Ischemic heart disease remains one of the leading causes of death worldwide. An elevated level of plasma cholesterol has been demonstrated to play an important role in the development of this fatal disease.[1] Recent large clinical studies have lead to the lower targets in plasma cholesterol levels (i.e., LDL cholesterol of <100 mg/dL) for patients with increased risk for coronary heart disease, which significantly increased the number of patients requiring cholesterol-lowering treatment.[1,2] Competitive inhibitors of 3-hydroxy-3-methylglutaryl coenzyme A (HMG-CoA) reductase (statins) are the gold standard in the treatment of hypercholesterolemia. However, since many patients do not

Correspondence to: Pavel Gershkovich (Telephone: 1-604-822-6772; Fax: 1-604-822-3035; E-mail: pgershko@interchange.ubc.ca)

Journal of Pharmaceutical Sciences, Vol. 98, 2390–2400 (2009)
© 2008 Wiley-Liss, Inc. and the American Pharmacists Association

WILEY InterScience® DISCOVER SOMETHING GREAT

respond adequately to statins therapy[3] and some develop a life-threatening side effects,[4–6] there is a strong need for alternative/adjuvant treatments of hypercholesterolemia.

The LDL cholesterol pool is derived from two major sources: endogenous synthesis and intestinal absorption. It was reported that in humans cholesterol absorption efficiently regulates plasma cholesterol levels.[7] The western diet provides in average 300–500 mg/day of cholesterol and bile contributes about 800–1200 mg/day. Since the inhibition of cholesterol absorption is associated with a compensatory rise in endogenous cholesterol synthesis,[8] it is conceivable that reduction in cholesterol absorption therapy should be combined with agents that decrease cholesterol synthesis. There is a long history of use of cholesterol absorption inhibitors for treating hypercholesterolemia.[1] The main agents are ezetimibe, plant sterols, and plant stanols. Ezetimibe is a highly potent and selective agent which inhibits the active uptake of cholesterol across the apical membrane of enterocyte.[1,9,10] The effect of ezetimibe is additive to statins and a single daily dose of 10 mg produces a 14% reduction in LDL cholesterol levels.[11,12] Although ezetimibe seems to be a relatively safe alternative option for hyperlipidemic patients, it is a new medication, and thus, should be used with caution.[13] In addition, it appears to reach a saturation effect on the reduction of cholesterol adsorption. Plant sterols and stanols are structurally similar to cholesterol. These agents are in a wide use as food additives to reduce plasma cholesterol levels.[14–16] The dose of 1.5–3 g/day leads to 8–16% decrease in plasma LDL cholesterol levels.[1] However, there are reports in the literature that even moderately increased plasma plant sterol levels might actually contribute to an increased risk for coronary heart disease.[17–19] Thus, there is a need for the development of alternative nonabsorbable agents for inhibition of intestinal absorption of cholesterol that would not have systemic adverse effects of plant sterols.

Potential candidates for nonabsorbable agents for inhibiting gastrointestinal absorption of cholesterol would be the naturally occurring aluminosilicate clays. The specific aluminosilicate clays examined in this report are known as montmorillonite minerals and commonly referred to as bentonite clays. Other examples of this chemically related aluminosilicates are saponite, beidellite, and nontronite. Montmorillonite is a layered aluminosilicate with the property of adsorbing both water and organic substances on its external surfaces or within its interlaminar space.[20] A schematic presentation of montmorillonite structure is shown in Figure 1. The montmorillonite clays are fine particle sized hydrous aluminosilicate with a platelet structure. The platelets are composed of three layers with the first and third layer being silicon oxygen tetrahedral layers and the middle layer composed primarily of aluminum

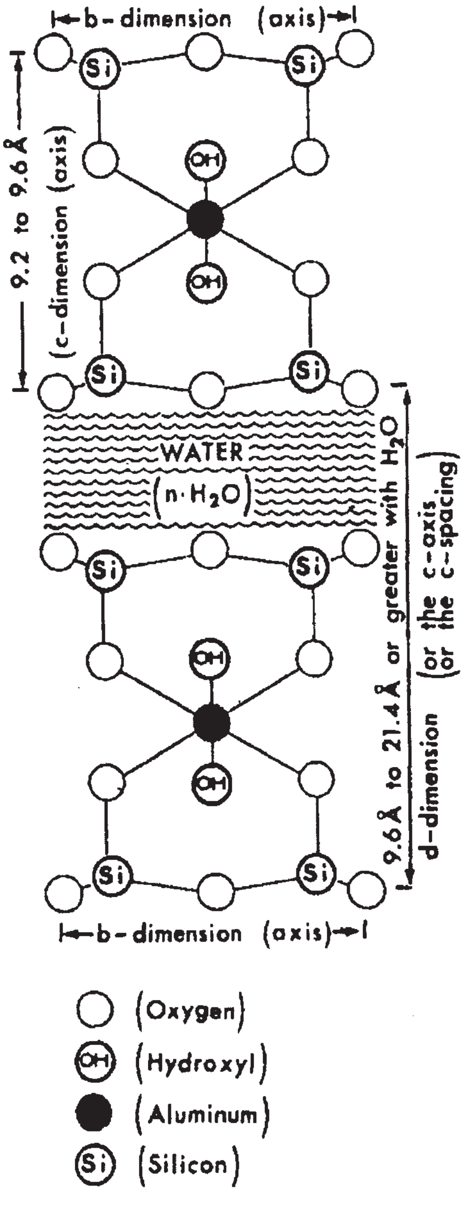

Figure 1. A schematic presentation of montmorillonite structure. Montmorillonite is a layered silicate with the property of adsorbing organic substances on its external surfaces or within its interlaminar space. The hydration of the clay induces swelling, which is mostly attributed to the increase in d (or c) dimension.

octahedral layers. The platelet dimensions are typically <1 nm in height and 200–400 nm in length and width. Adsorption properties of montmorillonite are attributed to its large specific surface area and high cation exchange capacity.[21] The clay platelets are not totally rigid but have some degree of flexibility and are indeed nanoparticulate. Thus, bentonite clays in general are considered nanostructured materials and the term nanoclays quite often appear in the literature. Within the content of the studies reported here, nanostructured aluminosilicates (NSAS) are used to reflect the nanodimensions of their structure and not the evaluated aqueous suspensions formed upon hydration of these materials and the formation of micro-sized particles. Other examples of this chemically related aluminosilicates are saponite, beidellite, and nontronite.

The dietary consumption of clay, including montmorillonite, has been well documented for centuries in animals and humans.[22] Montmorillonite clay has been evaluated for chronic consumption safety in rats[23] and was assessed for a short-term safety profile in humans.[24] It was found to be a relatively safe compound for both animals and humans. Due to its adsorption properties, montmorillonite has been used as anticaking additive in animal feed.[24] For the prevention of aflatoxicosis, bentonite clays are routinely added to animal feed to sorb aflatoxins in the gastrointestinal tract and diminish their absorption and adverse effects.[25] It was also shown to ameliorate hyperthyroidism in rats due to adsorption of thyroid hormone transferred to intestine in bile.[20] In humans, montmorillonite has been shown to be effective in the treatment of diarrhea[26] and in constipation-predominant irritable bowel syndrome.[27] In addition, montmorillonite has been used for the removal of heavy metal contaminants from water,[28] in cosmetics/dermatology,[29–31] and as a filler in pharmaceuticals.[32–35]

There are ancillary reports of montmorillonite being effective as a cholesterol-lowering aid and various smectite clays are sold in health food stores. However, although there are some limited reports from the 1950s on the adsorption of cholesterol to bentonite *in vitro*[36] and on a hypocholesterolemic effect of a food-grade silicon dioxide in rats,[37] there are no specific reports and publications on the effect of natural bentonite clay and surface-modified NSAS materials on intestinal cholesterol absorption in animals and/or humans.

The purpose of this work was to assess the ability of novel surface-modified NSAS compounds to inhibit the intestinal absorption of dietary cholesterol. The effect produced by NSAS compounds following oral administration to rats was compared to two cholesterol absorption inhibitors that are currently in clinical use, stigmastanol and ezetimibe.

MATERIALS AND METHODS

Materials

Cholesterol, stigmastanol, 10% Intralipid®, carboxymethyl cellulose, 6 N hydrochloric acid, and concentrated sodium hydroxide (Sigma–Aldrich, Oakville, ON, Canada) were all used as received. [^{3}H]Cholesterol was purchased from GE Healthcare (Mississauga, ON, Canada). Ezetimibe 10 mg tablets (Ezetrol®; Merck Frosst-Schering Pharma, Kirkland, Quebec, Canada) were received as a free sample. Ketamine HCL 100 mg/mL (Ketaset®; Wyeth Canada, St. Laurent, Quebec, Canada), and pentobarbital sodium 240 mg/mL (Euthanyl®; Bieda-MTC Animal Health Inc., Cambridge, ON, Canada) were purchased from McGill University, DRSS Animal Resource Centre (Montreal, Quebec, Canada). The proton ion-exchange resin was Amberlite FPC23 H available from Rohm & Hass (Philadelphia, PA). All other chemicals were of analytical reagent grade, and solvents were of HPLC grade.

Preparation of NSAS Compounds

Base NSAS

The crude NSAS was mined in Wyoming, United States and dried to 10% moisture content. The particle size was reduced by a passing through a 200 mesh (74 μm) screen. The resultant colloidal particles served as a base for the preparation of all NSAS compounds.

Nonpurified NSAS

The base NSAS was mixed with deionized water using a blender at 11,500 rpm for 5 min. The final sample contained 2.6% of solids (w/v).

Sodium NSAS

The base NSAS was purified by a previously reported method[38] and mixed with filtered deionized water using a blender at 11,500 rpm for 5 min. As a result of the purification process

essentially all of the exchangeable surface cations were replaced by sodium ions.[38] The final sample contained 2.5% of solids (w/v).

Hydrochloric Acid-Treated NSAS

Sodium NSAS sample was treated with 6 N HCl drop wise until a pH 2.2 was obtained. The final sample contained 2.8% of solids (w/v).

Protonated (by Ion Exchange) NSAS

Sodium NSAS sample was pumped through two lab-scale ion-exchange columns filled with hydrogen-loaded resin exchange beads to protonate the montmorillonite. The final sample contained 2.7% of solids (w/v).

Back-Alkanized (by NaOH) NSAS

A slurry sample of protonated (by ion exchange) NSAS was treated with 1 N NaOH drop wise until a pH 10 was obtained. Final mixture had 2.7% of solids (w/v).

All samples were gamma-irradiated following the preparation.

Characterization of NSAS Compounds

All characterization measurements were performed 24 h after the samples were prepared.

The pH was measured using an Oakton pH 110 series probe. The probe was placed in suspension sample and allowed to stabilize for at least 2 min before recording the pH.

Solids content of the suspension was measured at 110°C on a Mettler Toledo HB43 Halogen moisture balance.

Viscosity was measured using a Brookfield LVTD at 6 rpm. A 500 mL beaker was filled to the 400 mL mark with suspension sample. The spindle was allowed to rotate for 5 min in the sample before taking a measurement.

Zeta potential measurements were taken using a Malvern Nano-ZS zetasizer. A 20× dilution of each suspension sample was made by diluting with deionized water and mixing in a blender for 5 min. The diluted samples were then measured for zeta potential.

Particle size was measured using a Coulter LS230 particle size analyzer at 75% circulation speed. The particle size distribution was calculated using the Fraunhofer model. Each sample was measured in two ways. First, samples were measured as is without diluting or dispersing. Each sample was then diluted 1:10 and mixed in kitchen blender for 5 min at 7000 rpm and measured again for particle size. This was done to break-up any gelling that occurred with some of the samples.

Preparation of Formulation of Cold Cholesterol and [^{3}H]Cholesterol in 10% Intralipid®

The commercially available 20% (triglyceride concentration, v/v) Intralipid® was diluted by external phase of emulsion to reach triglyceride concentration of 10%. Cold cholesterol was dissolved in absolute ethanol (1 mg/75 μL) and the solution was slowly added to 10% Intralipid® with magnetic stirring. [^{3}H]Cholesterol toluene solution (25 μL/mL) was then slowly added and the formulation was vortex mixed for 30 s. Mixing by magnetic stirring was continued for additional 30 min until ready for administration. The final formulation contained 90% of Intralipid®, 7.5% of ethanol, and 2.5% of toluene (v/v). The final concentrations of cold and radiolabeled cholesterol were 1 mg/mL and 25 μCi/mL, respectively.

Preparation of the Ezetimibe Formulation

Commercially available Zetia® tablets were crushed and suspended in double distilled water (DDW) to reach final ezetimibe concentration of 5 mg/mL. The pharmaceutical excipients that are present in the commercially available tablets facilitate the formation of stable suspension. The suspension was briefly vortexed and then stirred using a magnetic stirrer for about 1 h until ready for administration.

Preparation of the Stigmastanol Formulation

Stigmastanol was prewetted by Tween 80 (1%, v/v, final concentration) and suspended in 2% sodium carboxymethyl cellulose solution in DDW. The suspension was briefly vortexed for about 3 min, sonicated (Bransonic® 3510) for 2 h at ambient temperature, and then stirred with a magnetic stirrer for 30 min until ready for administration. The final concentration of stigmastanol in the suspension was 25 mg/mL.

Surgery

All animals used in this study were cared for in accordance with the principles promulgated by the Canadian Council in Animal Care and the University of British Columbia. Male Sprague–Dawley rats (UBC Animal Care Center, Vancouver, BC, Canada) weighing approximately 350 g were used in this study. The rats were kept under a 12-h light/dark cycle with free access to water and food (regular rat chow).

To enable blood sampling, the right external jugular vein was cannulated with a two-part catheter consisting of PE-50 connected to silastic tubing. The surgery was performed under general anesthesia induced by intraperitoneal injection of 90 mg/kg of ketamine and 9 mg/kg of xylazine. The cannula was exteriorized at the dorsal part of the neck, which allowed carrying out the investigation in nonanesthetized and unrestrained rats. Following the surgery, animals were housed individually and allowed to recover for 24 h until the experiment.

Experimental Design

The solid food was withheld but a free access to oral liquid cholesterol-free diet consisting of 5% glucose in lactated Ringer's was allowed following the surgery and throughout the experiment. Twenty-four hours postsurgery, animals were divided into the following 10 treatment groups: nonpurified NSAS 50 mg/kg; sodium NSAS 50 mg/kg; protonated (by ion-exchange column) NSAS 20, 50, and 100 mg/kg; hydrochloric acid-treated NSAS 50 mg/kg; back-converted to sodium form (by NaOH) NSAS 50 mg/kg; stigmastanol 50 mg/kg; ezetimibe 10 mg/kg; and normal saline (control). All compounds were administered by oral gavage. Immediately following the first administration, animals in all groups received an additional oral gavage of radiolabeled formulation of cholesterol in Intralipid® in the dose of 1 mg of cholesterol per 350 g body weight (2.86 mg/kg). Systemic blood (0.35 mL) was sampled at 10 min predose and 0.5, 1, 2, 4, 6, 10, 24, 28, 32, and 48 h postdose. The withdrawn blood was replaced by intravenous bolus injection of equal volume of normal saline immediately following each blood sampling. Plasma was separated by centrifugation (5000 rpm, 10 min) and stored at -20°C until analysis. The concentration of administered cholesterol in plasma was determined against external calibration curves by means of radioactivity and expressed as ng/mL equivalent. It was previously shown (by thin-layer chromatography) that >90% of radioactivity counts in plasma following oral administration of [^{3}H]cholesterol to rats were associated with either esterified or unesterified cholesterol.[39] The area under curve (AUC) of plasma concentration–time profiles was calculated by trapezoidal method using WinNonlin 5.0.1 Professional software (Pharsight Corporation, Mountain View, CA).

Statistical Analysis

The data are presented as mean $\pm$ SEM, if not specified otherwise. The statistical significance of the differences between groups was determined using one-way ANOVA followed by Tukey–Kramer or Bonferroni posttests or using unpaired *t*-test with Welch correction, where appropriate. A p-value of <0.05 was considered statistically significant.

RESULTS AND DISCUSSION

Physico-Chemical Properties of NSAS Compounds

The physico-chemical properties of the suspensions of the investigated NSAS compounds are described in Table 1. It can be seen that there are significant differences between the samples in their pH, particle size before dilution as well as viscosity values (but not in particle size after dilution and zeta potential). It appears that the high viscosity of the protonated by ion-exchange column NSAS suspension may play a role in its efficacy in inhibiting the absorption of intestinal cholesterol (described below) by reducing its diffusion in the GI tract and/or disturbing bile salt metabolism and micelle formation. Consistent with this mechanism are the findings by Carr et al.[40] that increased intestinal contents viscosity upon feeding hamsters with hydroxypropylmethylcellulose (HPMC) reduced cholesterol absorption efficiency. However, additional studies are required in order to establish correlations between the viscosity and other physico-chemical properties of NSAS suspensions and their efficacy in inhibiting cholesterol absorption.

This research utilized two distinct methods to protonate the surface of the montmorillonite clay. The first method was the ion-exchange column and the second method was the addition of bulk mineral acid to the solution. It is expected that the

Table 1. Physico-Chemical Properties of the Suspensions of the NSAS Compounds

NSAS Compound	pH of Administered Sample[a] ($n = 5$)	Particle Size[b] (μm, $n = 4$)	Diluted 1:10 Particle Size[c] (μm, $n = 6$)	Zeta Potential[c] (mV, $n = 6$)	Brookfield Viscosity[d] (cPs, $n = 7$)
HCL acid treated NSAS	2.8 ± 0.04	2.68 ± 1.45	1.92 ± 0.74	-38.6 ± 2.1	3110 ± 53
Back alkalinized (by NaOH) NSAS	10.0 ± 0.08	42.02 ± 10.57	9.38 ± 3.67	-41.8 ± 4.4	800 ± 76
Nonpurified NSAS	9.1 ± 0.04	13.21 ± 7.84	10.96 ± 5.09	-36.8 ± 2.8	750 ± 57
Purified sodium NSAS	10.0 ± 0.13	1.44 ± 0.40	1.36 ± 0.42	-45.9 ± 2.6	670 ± 87
Protonated (by ion exchange) NSAS	2.6 ± 0.04	23.35 ± 4.54	10.41 ± 3.88	-46.1 ± 1.3	8300 ± 302

[a]Groups are statistically significantly different from each other except HCL acid treated versus protonated (by ion exchange) and except back alkalinized versus purified sodium NSAS.
[b]Statistically significantly different from each other are: HCL acid treated versus back alkalinized, back alkalinized versus nonpurified, and back alkalinized versus purified sodium NSAS.
[c]Differences between groups are statistically not significant.
[d]Groups are statistically significantly different from each other except back alkalinized versus nonpurified, back-alkalinized versus purified sodium, and nonpurified versus purified sodium NSAS.

ion-exchange column would exchange the proton for the sodium directly on the platelet surface whereas the bulk addition of mineral acid may not exchange all of the surface sodium ions. Thus, it is possible that we have merely acidified the solution while leaving the sodium ions on the surface of the platelet. Another important factor is by adding the bulk HCL, the ionic salt concentration of the suspension has significantly increased whereas in the ion-exchange column, the salt concentration has remained constant. In addition, there are reports in the literature that aggressive acid treatment can actually induce dissolution of different clay minerals, including montmorillonites.[42] This is also supported by surprisingly small particle size in this preparation in comparison to NSAS protonated by means of an ion-exchange column (Tab. 1).

The Plasma-Concentration Profiles of Radioactive Cholesterol Following its Oral Administration With Tested Compounds

The plasma concentration–time profiles of radioactive cholesterol (expressed as ng/mL equivalents) following administration of cholesterol with NSAS compounds, ezetimibe or stigmastanol (positive controls), or normal saline (negative control) are shown in Figure 2. It could be seen that the magnitude of the exposure but not the shape of the profiles is affected by different treatments, which indicates that the observed changes are related primarily to the absorption phase rather than to subsequent distribution or elimination. This is not surprising since NSAS compounds are not absorbed following oral administration and their effects are localized within the intestinal lumen. It should be noted that the mechanisms of action of both positive controls, ezetimibe and stigmastanol, are also linked to the intestinal absorption phase of cholesterol homeostasis.

Effect of Protonation of Aluminosilicate on its Efficacy in Regards to Inhibition of Intestinal Cholesterol Absorption

The $AUC_{0–48\ h}$ calculated from plasma concentration–time profiles of normal saline group (vehicle control), and groups at 50 mg/kg of the following NSAS compounds: nonpurified material, sodium form, protonated (by ion-exchange column), hydrochloric acid-treated, and back-converted (by NaOH) to sodium form are presented in Figure 3. There is a statistically significant ($p < 0.05$) $31.5 \pm 18.9\%$ reduction in cholesterol absorption in rats that received 50 mg/kg of protonated (by ion-exchange column) NSAS ($AUC_{0–48\ h} = 55{,}400 \pm 7555$ h ng/mL) comparing to the control animals ($AUC_{0–48\ h} = 80{,}861 \pm 5911$ h ng/mL). On the other hand, no significant differences were found between the control animals and rats that received the same dose (50 mg/kg) of nonpurified material ($AUC_{0–48\ h} = 79{,}744 \pm 4707$ h ng/mL) or sodium form ($AUC_{0–48\ h} = 70{,}718 \pm 9019$ h ng/mL). Moreover, conversion of the protonated form back to

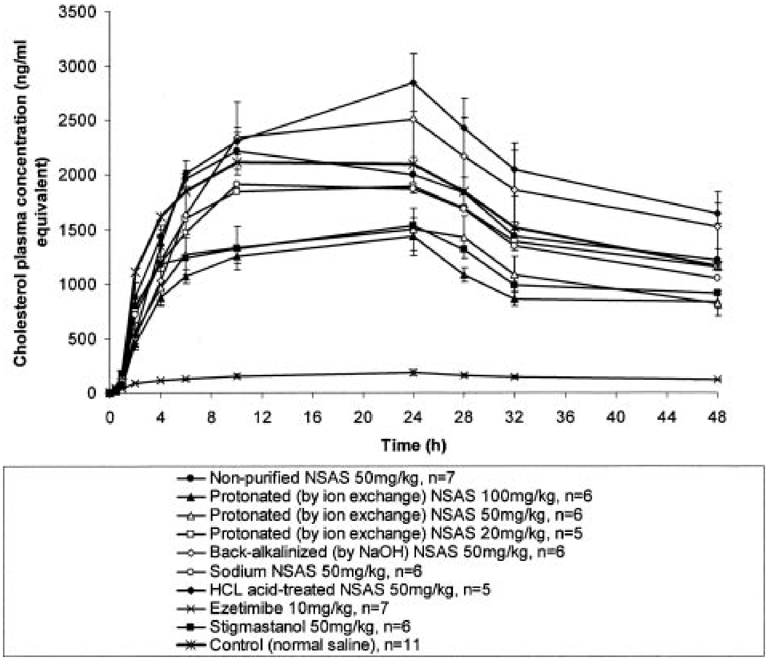

Figure 2. The plasma concentration–time profiles of radioactive cholesterol (expressed as ng/mL equivalents, mean ± SEM) following oral administration of cholesterol with tested compounds or with normal saline (negative control).

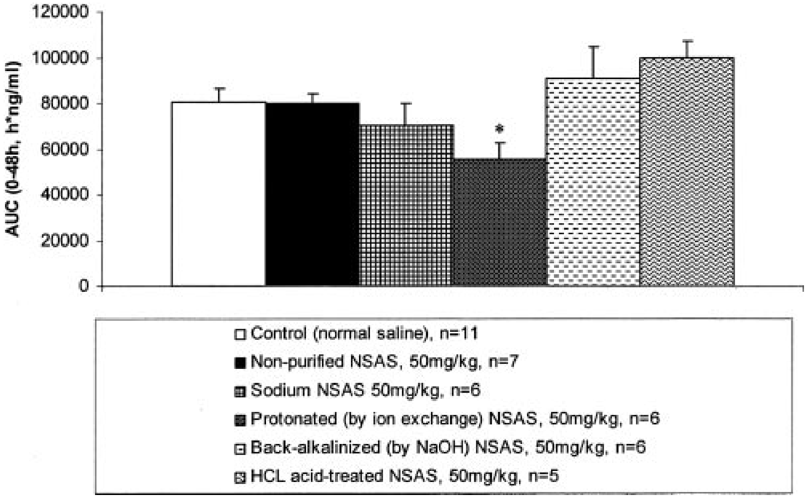

Figure 3. The area under curve values (mean ± SEM) calculated from plasma concentration–time profiles of radiolabeled cholesterol following oral administration with normal saline or with 50 mg/kg of either: nonpurified NSAS, sodium form of NSAS, protonated (by ion-exchange column) NSAS, back-alkalinized NSAS or with acid-treated NSAS. Only protonated (by ion-exchange column) NSAS material demonstrated statistically significant inhibition in cholesterol absorption ($p < 0.05$, one-way ANOVA followed by Bonferroni posttest) relatively to control group when administered orally in the dose of 50 mg/kg. All other NSAS compounds, administered at the same dose, have not demonstrated statistically significant effect on the intestinal absorption of cholesterol.

sodium form by NaOH treatment resulted in loss of the effect of NSAS material on cholesterol absorption ($AUC_{0-48\,h} = 90{,}992 \pm 13{,}460$ h ng/mL). These results suggest that protonation of NSAS by means of ion-exchange column increases its efficacy in regards to inhibition of intestinal cholesterol absorption by direct and/or indirect mechanisms. In reference to direct effects and by taking into consideration previous reports on the adsorption of cholesterol molecules to bentonite clay,[36] it may be possible that protonated NSAS reduce the extent of cholesterol absorption by direct adsorption of intestinal cholesterol. However, it should be noted that other mechanisms are possible under *in vivo* conditions, such as adsorption of bile acids, fatty acids, monoglycerides, or other constituents of cholesterol-containing mixed micelles to the NSAS or alteration in the lipolysis process of coadministered lipid-based formulation.

It is unclear at this stage whether or not the inhibition of dietary cholesterol absorption by protonated NSAS will result in significant reduction in plasma cholesterol levels in hypercholesterolemic patients. In our preliminary short-term study (5 weeks) in the hypercholesterolemic rabbits model, protonated surface-modified material induced small but consistent reduction in plasma LDL-cholesterol levels.[41] Since the number of animals in that study was too small to show statistical significance, longer chronic study in Apo-E deficient mice is underway in order to assess the possible utilization of this agent for the treatment of hypercholesterolemia.

Interestingly, an attempt to protonate NSAS by hydrochloric acid failed in regards to the efficacy of the compound in inhibiting cholesterol absorption ($AUC_{0-48\,h} = 99{,}511 \pm 7415$ h ng/mL). Incomplete protonation and/or major platelet exfoliation and structural changes in the clay matrix are likely reasons for the lack of cholesterol absorption inhibition activity of hydrochloric acid-treated NSAS.

The Dependence of the Degree of Inhibition of Cholesterol Absorption upon the Dose of Protonated NSAS

Figure 4 presents the dependence of exposure to radioactive cholesterol ($AUC_{0-48\,h}$) on the dose of administered protonated (by ion-exchange column) NSAS clay 0 mg/kg (control), 20, 50, and 100 mg/kg. Administration of both 50 and 100 mg/kg ($AUC_{0-48\,h} = 49{,}609 \pm 2090$ h ng/mL) of protonated NSAS (but not 20 mg/kg, $AUC_{0-48\,h} = 72{,}227 \pm 10{,}560$ h ng/mL) resulted in statistically significant decrease in cholesterol absorption relatively to the control animals. It can be seen that as the dose of protonated NSAS is increased,

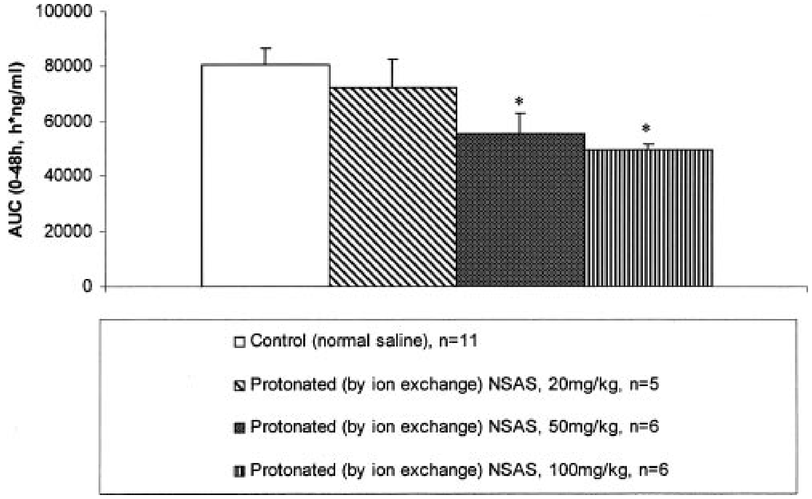

Figure 4. The area under curve values (mean ± SEM) calculated from plasma concentration–time profiles of radiolabeled cholesterol following oral administration with normal saline or with protonated NSAS 20, 50, and 100 mg/kg. Although the difference between different doses was not statistically significant, both 50 and 100 mg/kg (but not 20 mg/kg) doses resulted in statistically significant inhibition of intestinal cholesterol absorption ($p < 0.05$, one-way ANOVA with Tukey–Kramer posttest).

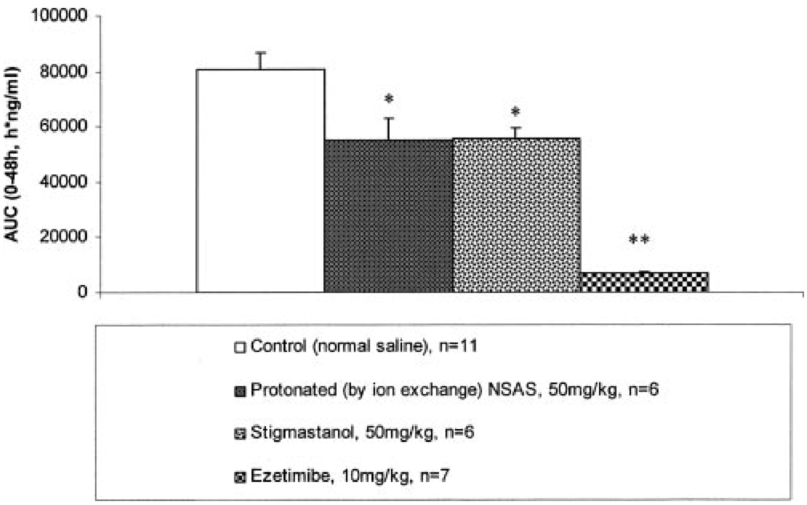

Figure 5. The area under curve values (mean $\pm$ SEM) calculated from plasma concentration–time profiles of radiolabeled cholesterol following oral administration with normal saline, protonated NSAS 50 mg/kg, stigmastanol 50 mg/kg, or ezitimibe 10 mg/kg. Treatments by protonated NSAS material or by stigmastanol resulted in statistically significant inhibition in cholesterol absorption ($p < 0.05$, one-way ANOVA with Bonferroni posttest), while there is no difference between these two compounds. Treatment by ezetimibe 10 mg/kg resulted in extremely significant decrease in exposure to radiolabeled cholesterol relatively to the control group ($p < 0.01$, unpaired t-test with Welch correction).

there is a trend of an increase in the effect, while the variability in response to treatment is decreased. However, although there is a continued trend to decrease in cholesterol absorption when the dose is increased from 20 to 50 mg/kg and then to 100 mg/kg, this increase in effect between groups (dose dependence) was not found to be statistically significant in this study.

Comparison of Protonated NSAS to Cholesterol Absorption Inhibitors in Clinical Use

Figure 5 represents the comparison of exposure to radiolabeled cholesterol when it is given with normal saline (vehicle control), 50 mg/kg of protonated NSAS, 50 mg/kg of stigmastanol ($AUC_{0\text{–}48\ h} = 55{,}742 \pm 3764$ h ng/mL), or with ezetimibe 10 mg/kg ($AUC_{0\text{–}48\ h} = 6937 \pm 462$ h ng/mL). Both protonated NSAS and stigmastanol induce the same statistically significant inhibition in intestinal cholesterol absorption relatively to the control group. Treatment by ezetimibe 10 mg/kg resulted in higher inhibition of intestinal cholesterol absorption than protonated NSAS 50 mg/kg or stigmastanol 50 mg/kg.

CONCLUSIONS

It was found in this study that surface-modified NSAS material by ion-exchange protonation effectively inhibits the intestinal absorption of dietary cholesterol in a rat model. Although direct interaction of NSAS materials with bile salts and/or cholesterol is a possibility, indirect mechanisms involving changes in the intestinal contents viscosity by the administered NSAS cannot be ruled out. Further studies are needed for the elucidation of the mechanism(s) by which protonated forms of NSAS materials inhibit the intestinal absorption of dietary cholesterol. Additional studies are underway to assess whether or not the observed inhibition of intestinal absorption of dietary cholesterol will result in a significant reduction of plasma cholesterol levels in hypercholesterolemic animals upon chronic administration of protonated NSAS material.

ACKNOWLEDGMENTS

This study was funded by AMCOL International Corporation.

REFERENCES

1. Wang DQ. 2007. Regulation of intestinal cholesterol absorption. Annu Rev Physiol 69:221–248.
2. Grundy SM, Cleeman JI, Merz CN, Brewer HB, Jr., Clark LT, Hunninghake DB, Pasternak RC, Smith SC, Jr., Stone NJ. 2004. Implications of recent clinical trials for the National Cholesterol Education Program Adult Treatment Panel III guidelines. Arterioscler Thromb Vasc Biol 24:149–161.
3. Kruit JK, Groen AK, van Berkel TJ, Kuipers F. 2006. Emerging roles of the intestine in control of cholesterol metabolism. World J Gastroenterol 12: 6429–6439.
4. Harper CR, Jacobson TA. 2007. The broad spectrum of statin myopathy: From myalgia to rhabdomyolysis. Curr Opin Lipidol 18:401–408.
5. Davidson MH, Robinson JG. 2007. Safety of aggressive lipid management. J Am Coll Cardiol 49:1753–1762.
6. Kashani A, Phillips CO, Foody JM, Wang Y, Mangalmurti S, Ko DT, Krumholz HM. 2006. Risks associated with statin therapy: A systematic overview of randomized clinical trials. Circulation 114:2788–2797.
7. Kesaniemi YA, Miettinen TA. 1987. Cholesterol absorption efficiency regulates plasma cholesterol level in the Finnish population. Eur J Clin Invest 17:391–395.
8. Santosa S, Varady KA, AbuMweis S, Jones PJ. 2007. Physiological and therapeutic factors affecting cholesterol metabolism: Does a reciprocal relationship between cholesterol absorption and synthesis really exist? Life Sci 80:505–514.
9. Huff MW, Pollex RL, Hegele RA. 2006. NPC1 L1: Evolution from pharmacological target to physiological sterol transporter. Arterioscler Thromb Vasc Biol 26:2433–2438.
10. Patel SB. 2004. Ezetimibe: A novel cholesterol-lowering agent that highlights novel physiologic pathways. Curr Cardiol Rep 6:439–442.
11. Bitzur R, Cohen H, Harats D. 2006. Cholesterol absorption as a target for the treatment of hypercholesterolemia. Harefuah 145:831–835, 860, 861.
12. Wierzbicki AS. 2004. Lipid-altering agents: The future. Int J Clin Pract 58:1063–1072.
13. Florentin M, Liberopoulos EN, Elisaf MS. 2008. Ezetimibe-associated adverse effects: What the clinician needs to know. Int J Clin Pract 62:88–96.
14. Maki KC, Davidson MH, Umporowicz DM, Schaefer EJ, Dicklin MR, Ingram KA, Chen S, McNamara JR, Gebhart BW, Ribaya-Mercado JD, Perrone G, Robins SJ, Franke WC. 2001. Lipid responses to plant-sterol-enriched reduced-fat spreads incorporated into a National Cholesterol Education Program Step I diet. Am J Clin Nutr 74:33–43.
15. Miettinen TA, Puska P, Gylling H, Vanhanen H, Vartiainen E. 1995. Reduction of serum cholesterol with sitostanol-ester margarine in a mildly hypercholesterolemic population. N Engl J Med 333: 1308–1312.
16. Ostlund RE, Jr., Racette SB, Okeke A, Stenson WF. 2002. Phytosterols that are naturally present in commercial corn oil significantly reduce cholesterol absorption in humans. Am J Clin Nutr 75:1000–1004.
17. Sudhop T, von Bergmann K. 2004. Sitosterolemia—A rare disease. Are elevated plant sterols an additional risk factor? Z Kardiol 93:921–928.
18. Patel MD, Thompson PD. 2006. Phytosterols and vascular disease. Atherosclerosis 186:12–19.
19. Assmann G, Cullen P, Erbey J, Ramey DR, Kannenberg F, Schulte H. 2006. Plasma sitosterol elevations are associated with an increased incidence of coronary events in men: Results of a nested case-control analysis of the Prospective Cardiovascular Munster (PROCAM) study. Nutr Metab Cardiovasc Dis 16:13–21.
20. Cai Y, Meng XF, Cao YX, Lu H, Zhu SF, Zhou LZ. 2006. Montmorillonite ameliorates hyperthyroidism of rats and mice attributed to its adsorptive effect. Eur J Pharmacol 551:156–161.
21. Ma YL, Guo T. 2008. Intestinal morphology, brush border and digesta enzyme activities of broilers fed on a diet containing Cu^{2+}-loaded montmorillonite. Br Poult Sci 49:65–73.
22. Carretero MI. 2002. Clay minerals and their beneficial effects upon human health. Appl Clay Sci 21: 155–163.
23. Afriyie-Gyawu E, Mackie J, Dash B, Wiles M, Taylor J, Huebner H, Tang L, Guan H, Wang JS, Phillips T. 2005. Chronic toxicological evaluation of dietary NovaSil clay in Sprague-Dawley rats. Food Addit Contam 22:259–269.
24. Wang JS, Luo H, Billam M, Wang Z, Guan H, Tang L, Goldston T, Afriyie-Gyawu E, Lovett C, Griswold J, Brattin B, Taylor RJ, Huebner HJ, Phillips TD. 2005. Short-term safety evaluation of processed calcium montmorillonite clay (NovaSil) in humans. Food Addit Contam 22:270–279.
25. Phillips TD, Lemke SL, Grant PG. 2002. Characterization of clay-based enterosorbents for the prevention of aflatoxicosis. Adv Exp Med Biol 504: 157–171.
26. Madkour AA, Madina EM, el-Azzouni OE, Amer MA, el-Walili TM, Abbass T. 1993. Smectite in acute diarrhea in children: A double-blind placebo-controlled clinical trial. J Pediatr Gastroenterol Nutr 17:176–181.
27. Ducrotte P, Dapoigny M, Bonaz B, Siproudhis L. 2005. Symptomatic efficacy of beidellitic montmorillonite in irritable bowel syndrome: A randomized, controlled trial. Aliment Pharmacol Ther 21:435–444.
28. Bhattacharyya KG, Gupta SS. 2008. Adsorption of a few heavy metals on natural and modified kaolinite and montmorillonite: A review. Adv Colloid Interface Sci 140:114–131.

29. Sternberg TH, Taylor DR. 1951. Fused bentonite sulphur as a topical medication. Urol Cutan Rev 55:348–352.
30. Saary J, Qureshi R, Palda V, DeKoven J, Pratt M, Skotnicki-Grant S, Holness L. 2005. A systematic review of contact dermatitis treatment and prevention. J Am Acad Dermatol 53:845.
31. Elmore AR. 2003. Final report on the safety assessment of aluminum silicate, calcium silicate, magnesium aluminum silicate, magnesium silicate, magnesium trisilicate, sodium magnesium silicate, zirconium silicate, attapulgite, bentonite, Fuller's earth, hectorite, kaolin, lithium magnesium silicate, lithium magnesium sodium silicate, montmorillonite, pyrophyllite, and zeolite. Int J Toxicol 22: 37–102.
32. Viseras C, Lopez-Galindo A. 2000. Characteristics of pharmaceutical grade phyllosilicate powders. Pharm Dev Technol 5:47–52.
33. Puranik PK, Manekar NC, Dorle AK. 1992. Preparation and evaluation of abietic acid microcapsules by a solvent evaporation technique. J Microencapsul 9:425–435.
34. Kristensen J, Schaefer T, Kleinebudde P. 2002. Development of fast-disintegrating pellets in a rotary processor. Drug Dev Ind Pharm 28:1201–1212.
35. Meshali MM. 1982. Adsorption of phenazopyridine hydrochloride on pharmaceutical adjuvants. Pharmazie 37:718–720.
36. Nikkila E, Oker-Blom N. 1952. Effect of treatment of bentonite on serum protein, lipid phosphorus, and cholesterol. Ann Med Exp Biol Fenn 30:51–58.
37. Peluso MR, Schneeman BO. 1994. A food-grade silicon dioxide is hypocholesterolemic in the diet of cholesterol-fed rats. J Nutr 124:853–860.
38. Clarey M, Edwards J, Tsipursky SJ, Beall GW, Eisenhour DD. 2000. Method of manufacturing polymer-grade clay for use in nanocomposites. US Patent 6050509.
39. Wasan KM, Holtorf L, Subramanian R, Cassidy SM, Pritchard PH, Stewart DJ, Novak E, Moghadasian MH. 2001. Assessing plasma pharmacokinetics of cholesterol following oral coadministration with a novel vegetable stanol mixture to fasting rats. J Pharm Sci 90:23–28.
40. Carr TP, Gallaher DD, Yang CH, Hassel CA. 1996. Increased intestinal contents viscosity reduces cholesterol absorption efficiency in hamsters fed hydroxypropyl methylcellulose. J Nutr 126:1463–1469.
41. Sivak O, Gershkovich P, Darlington J, Constantinides P, Wasan KM. 2008. American Association of Pharmaceutical Scientists Annual Meeting and Exposition, Atlanta, GA, USA, November.
42. Madejova J, Bujdak J, Janek M, Komadel P. 1998. Comparative FT-IR study of structural modifications during acid treatment of dioctahedral smectites and hectorite. Spectrochimica Acta A 54:1397–1406.
